# Temporal trends in the incidence rates of keratinocyte carcinomas from 1978 to 2018 in Tasmania, Australia: a population-based study

**DOI:** 10.1007/s12672-021-00426-5

**Published:** 2021-08-31

**Authors:** Bruna S. Ragaini, Leigh Blizzard, Leah Newman, Brian Stokes, Tim Albion, Alison Venn

**Affiliations:** 1 Menzies Institute for Medical Research, University of Tasmania, Private Bag 23, Hobart, TAS 7000 Australia; 2 Australian Institute of Health and Welfare, Canberra, Australia; 3 Tasmanian Cancer Registry, Menzies Institute for Medical Research, University of Tasmania, Hobart, Australia

## Abstract

**Objectives:**

We described incidence trends of keratinocyte carcinomas (KCs)—namely basal cell carcinoma (BCC) and squamous cell carcinoma (SCC)—in the Australian state of Tasmania.

**Methods:**

We identified histologically confirmed KCs within the Tasmanian Cancer Registry (TCR) and conducted assessments to ensure data quality. Age-standardised incidence rates were calculated for first (1985–2018) and annual KCs (1978–2018). Average annual percentage changes were computed using Joinpoint regression models.

**Results:**

The TCR is a reliable source of KC data. A total of 83,536 people were registered with a KC between 1978 and 2018. Age-standardised incidence rates of first KCs increased on average by 3% per annum for BCCs and 4% per annum for SCCs, reaching 363/100,000 and 249/100,000 in 2018, respectively. Age-standardised incidence rates of annual KCs increased on average by 5% per annum for BCCs and 6% per annum for SCCs, up to 891/100,000 and 514/100,000 in 2018, respectively. This increase was steeper for females than males and highest during the late 1980s and early 1990s. A change in trend around 2014 suggested that incidence rates have started to decline.

**Conclusion:**

While the incidence of KCs in Tasmania increased substantially over 41 years, rates have recently plateaued and started to decline. The findings may reflect changes in sun exposure behaviours due to awareness campaigns, but high incidence rates in 2018 indicate that KCs still pose a substantial burden to this population.

**Supplementary Information:**

The online version contains supplementary material available at 10.1007/s12672-021-00426-5.

## Introduction

Skin cancer is the most common type of cancer in fair-skinned populations and Australia has the highest incidence in the world [1]. Known as keratinocyte carcinomas (KCs), basal cell carcinoma (BCC) and cutaneous squamous cell carcinoma (SCC) are the most prevalent cancers in Australia. Based on national survey data collected in 2002, the risk of being diagnosed by age 70 years was estimated at 70% for men and 58% for women [2]. With an age-standardised mortality rate of 1.9/100,000 (including rare types of non-melanoma skin cancers), KCs are rarely life threatening and account for only 1.2% of all cancer deaths [3]. However, the economic burden is substantial. KCs represented 8% of all cancer-related healthcare system costs in 2008–09 [4], $127.6 million over 959,243 benefits claimed in 2014 through Medicare—Australia’s publicly-funded universal healthcare insurance scheme—and 114,722 hospitalisations in 2013–14 [3].

Few population-based studies have examined trends in KC incidence in Australia [5]. The most reliable estimates used four national household surveys conducted between 1985 and 2002, which showed an increase in the age-standardised incidence from 657/100,000 to 884/100,000 BCCs and from 166/100,000 to 387/100,000 SCCs during the period [2, 6–8]. While these have contributed to knowledge, the surveys relied heavily on self-reported history of KCs, which can result in error [9, 10]. Further, the coverage, completeness and granularity of the data were limited, and the studies are now outdated. More recently, a study using Medicare claims for item numbers associated with the treatment of histologically confirmed KCs showed an increase of 86% in the total number of claims made between 1997 and 2010 [11]. However, Medicare claims do not differentiate between BCCs and SCCs, nor do they account for multiple claims for the same person, hindering our ability to examine trends in the two histological types separately and to separate counts of people from counts of claims. More granular and reliable trend data are needed for the evaluation of sun awareness campaigns and for the planning of healthcare services and resource allocation [3].

Population-based cancer registries provide the most reliable source of cancer incidence data in Australia, and these are regularly used to report cancer trends [12, 13]. However, KCs are typically not notifiable diseases by law and not routinely registered by cancer registries. As a result, KCs are excluded from routine state and national statistics reports and are only reported sporadically based on findings from surveys and administrative data collections. Only two previous studies have used registry data to estimate KC incidence in Australia, but these were conducted more than three decades ago in the state of Tasmania (1978–1987) [14] and the Northern Territory (1981–1985) [15].

Despite lacking a legislative basis, the Tasmanian Cancer Registry (TCR) has been notified of KCs diagnosed histologically in the state since 1978. However, manual registration ceased in 2005 due to lack of resources. Recently, an automated coding system was developed to facilitate the registration of notifications received from 2006 onwards, and a dataset from 1978 to 2018 was created. There are two aims to this study. First, to validate the dataset by assessing its accuracy and completeness. Second, to describe trends in the incidence rates of first and annual BCCs and SCCs in Tasmania over 41 years.

## Methods

### Study design and population

This population-based study included people identified in the TCR with a histologically confirmed diagnosis of BCC and/or SCC from January 1st, 1978 to December 31st, 2018.

Tasmania is an Australian island state located between 40ºS and 43ºS with approximately 534,457 residents [16] and the oldest population in Australia (median age = 42 years). Only 5% of Tasmanians identify as Aboriginal and/or Torres Strait Islander people, with the most common ancestry being European [17]. This study was approved by the Tasmanian Health and Medical Human Research Ethics Committee (reference H0018089).

The TCR has been routinely notified of histologically confirmed KC diagnoses by pathology laboratories, hospitals, and oncology clinics since its establishment in 1977. Initially, all KC lesions and their anatomical sites were registered by trained medical coders. However, due to the high costs associated with the manual registration of these common cancers, registration of KCs has been intermittent and ceased for notifications received after December 31st, 2005.

Recently, an automated coding system was developed using Visual Basic for Applications (VBA) to search electronic messages supplied by notifiers for keywords that may indicate the morphology and topography of the tumour, according to the convention of the International Classification of Diseases for Oncology, Third Edition (ICD-O3) [18]. Messages with keywords associated with skin sites (C44), morphology codes 805–808 and 809–811 and a fifth digit behaviour code three (malignant, primary) were ascertained as SCCs and BCCs, respectively, and those preceded by negation terms (e.g., ‘*no*’, ‘*raising the possibility*’, etc.) were excluded.

It is common for multiple KC lesions to be biopsied concurrently and reported under a single notification. However, the format in which this information is described in the notification varies widely from one pathologist to another, prohibiting the ability of the automated coding system to differentiate multiple lesions and accurately assign their anatomical sites. Consequently, only the first KC lesion of each histological type per notification is coded into the database and its anatomical site not reported. For instance, if a notification reported three BCCs and two SCCs, only the first BCC and the first SCC in the notification were coded.

Further, because the automated coding system cannot differentiate multiple lesions and correctly assign anatomical sites, we were unable to differentiate between biopsy samples and full excisions. Residual and recurrent lesions were excluded by using negation terms, but the same procedure was not appropriate for biopsy samples and full excisions because the former does not always precede the latter. As a result, the same lesion may be registered twice in the dataset. (See Statistical Analysis for how this was addressed).

The automated coding system reads all cancer notifications, then flags and codes those that report a confirmed KC diagnosis. All KC-related paper-based notifications received from January 1st, 2006 were scanned and digitised using OpenText™ TeleForm™ character recognition software, stored in a MS Access database, and processed and registered by the automated coding system. In mid-2012, paper-based notifications were replaced by Health Level 7 (HL7) electronic messages, which were stored in a SQL Server database and subsequently processed and registered by the automated coding system.

The manually (pre-2006) and automatically coded (2006 onwards) datasets were combined into a single MS Access database ranging from 1978 to 2018. Probabilistic data linkage was used to identify records belonging to the same person based on demographic information (name, date of birth, sex, and residential address at diagnosis). Clerical review was conducted manually to resolve pairs of records that were uncertain matches using additional information, such as previous names.

Two data quality assurance projects estimated the accuracy of registration and coding. The first occurred in 2005 when notifiers were audited for two four-week periods in 1995 and 2000. The months of March (end of summer) and September (end of winter) were selected as periods of typically high and low activity and represented 19% (n = 863) and 17% (n = 1,100) of all KC notifications for the two selected years, respectively. These notifications were compared with those already registered in the TCR database. More recently, we selected a sample of 1,000 TeleForm™-processed notifications (2006-mid 2012) and 1,500 HL7 messages (mid 2012–2018) and compared the automated registration and coding of KCs with a gold standard (i.e., manual registration and coding).

To assess the completeness of the dataset, we made several comparisons. First, notifications received during the audit were compared with those registered in the database to identify missing cases. Second, the total number of KC notifications registered by the TCR was compared with the claims made in Tasmania relating to the eight Medicare Benefit Scheme item numbers associated with the surgical excision of histologically confirmed KCs that were in effect between May 5th, 1997 and October 31st, 2016 (31,255, 31,260, 31,265, 31,270, 31,275, 31,280, 31,285 and 31,290). Third, estimated incidence rates of annual BCC and SCC (standardised to the 1960 World population [19]) were compared with the findings from previous national surveys that reported results for the Southern Australian latitudes above 37°S for the years 1985, 1990, 1995 and 2002 [2, 6–8, 20].

### Statistical analysis

The accuracy of registration and coding is reported as the numbers and percentages of records for which the results of manual coding matched that of automated coding by histological type and sex. The completeness of coding was assessed by making the comparisons described above.

To describe and assess trends in the incidence of KCs over the study period, age- and sex-specific incidence rates were estimated separately by histological type for first and annual notifications and standardised to the 2001 Australian standard population [21].

Incidence rates of both first and annual KCs were person-based (based on the number of persons diagnosed with a lesion). For incidence rates of first KCs, we counted only the first notification of BCC and SCC per person in the TCR between 1985 and 2018. This period was selected because the data showed that, among people who have more than one lesion notified, 75% of subsequent notifications occur within six years of the first notification. Therefore, we assumed that lesions registered from 1985 (at least 6 completed years after the commencement of registration) with no previous history of registration by the TCR were the first notification for a person. For incidence rates of annual KCs, we counted one notification of BCC and SCC per person each year for the entire period between 1978 and 2018.

Seventy-eight observations were excluded for missing data on date of diagnosis (n = 77) and sex (n = 1). Less than 0.5% of annual and first KCs belonged to people residing interstate at the time of diagnosis.

Because the automated coding system does not differentiate between biopsy samples and full excisions, we conducted a sensitivity analysis to examine changes in incidence rates of annual KCs after excluding registrations within three months (91 days) of an index notification. While the three-month length of period assumed for synchronicity is arbitrary, it has been used previously to minimise the registration of residual or recurrent cancers as new cancers [22, 23].

To assess temporal trends, incidence rates were analysed using the Joinpoint Regression Program [24] to estimate the average annual percentage change (AAPC) by sex, age, and histological type. A logarithmic transformation was selected, and the results are reported as percentage changes. Bayesian Information Criterion was used to select the optimal model, with a maximum of 5 joinpoints allowed.

The cumulative risk of being diagnosed with a first KC was calculated at ages 75, 80 and 85 years, by sex and histological type.

## Results

### Accuracy of registration and coding of KCs by the TCR

The audit found that 0.8% (n = 16) of notifications registered during the two four-week periods in 1995 and 2000 contained coding and data entry errors. These mostly resulted from misspellings of people’s names (18%), and incorrect morphological type (18%), body site (9%) and date of diagnosis (27%).

Compared with the gold standard of manual registration, the automated coding system misclassified less than 1% of BCC and 1% of SCC notifications (Online Resource 1). Misclassification was slightly higher in the sample of TeleForm™-processed notifications than HL7 messages due to less-than-optimal character recognition achieved during the scanning of paper-based notifications using TeleForm™.

### Completeness of KC registration by the TCR

During the audit, we identified 20 KC notifications that had not been received by the TCR, representing 2% and 0.5% of the total notifications in 1995 and 2000, respectively. Among these, 65% were BCCs and 74% were males. A further six notifications were received but not registered in the database, and information regarding the histological type and sex distribution of these missing cases was not recorded.

From 1997, the number of pathology notifications received by the TCR was slightly higher than the Medicare item number claims for the treatment of histologically confirmed KCs, a difference that increased after 2005 when KC registration became automated (Online Resource 2).

The age-standardised incidence rates of annual BCCs for Tasmania estimated using registry data were substantially lower than those estimated based on national surveys. Although similar between the two data sources, incidence rates of annual SCCs for Tasmania were sometimes higher or lower than estimates from the national surveys, which had wider confidence intervals due to the small sample of SCCs for Southern latitudes above 37°S (Online Resource 3).

### Trends in the incidence rates of first KCs

A total of 80,497 people were registered with a first KC between 1985 and 2018. Among them, 21% had both a BCC and a SCC. Of first notifications, 61% were BCCs and 39% were SCCs (Table 1).

**Table 1:** Characteristics of the study population

	Basal cell carcinoma^a^ % (n)	Squamous cell carcinoma^a^ % (n)
**First notifications**^b^		
Total (n)	59,337	38,043
Sex		
Male	54.7% (32,435)	57.0% (21,701)
Female	45.3% (26,902)	43.0% (16,342)
Age (years)		
Mean (SD)	63.6 (14.3)	70.0 (12.4)
Calendar period		
1985–1989	6.8% (4,023)	4.0% (1,530)
1990–1999	22.6% (13,407)	20.7% (7,874)
2000–2009	31.9% (18,896)	32.5% (12,344)
2010–2018	38.8% (23,011)	42.8% (16,295)
**Annual notifications**^c^		
Total (n)	126,083	68,744
Sex		
Male	60.1% (75,708)	61.0% (41,915)
Female	40.0% (50,375)	39.0% (26,829)
Age (years)		
Mean (SD)	66.7 (13.7)	72.6 (12.0)
Calendar period		
1978–1989	6.8% (8,549)	4.7% (3,215)
1990–1999	17.5% (22,110)	16.7% (11,491)
2000–2009	31.5% (39,662)	31.8% (21,831)
2010–2018	44.2% (55,762)	46.9% (32,207)

*SD* standard deviation

^a^Only 1 BCC and 1 SCC per notification are included

^b^Only first notification per person was included in the first counts, assuming that KCs registered from 1985 onwards without a previous history of registration by the Tasmanian Cancer Registry were the first ever KC notification for a person

^c^One notification per person per year was included in the annual counts

**Fig. 1:**
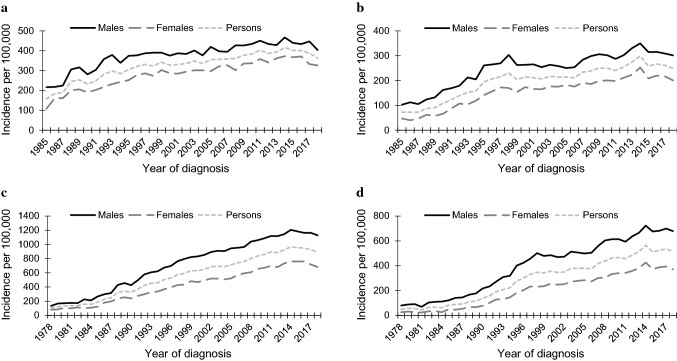
Temporal trends in the incidence rates of keratinocyte carcinoma in Tasmania, by histological type and sex. Incidence rates are standardised to the Australian 2001 population. **a** First basal cell carcinoma, 1985–2018; **b** first squamous cell carcinoma, 1985–2018; **c** annual basal cell carcinoma, 1978–2018; **d** annual squamous cell carcinoma, 1978–2018

There was an increase in the age-standardised incidence rates of first KCs in Tasmania between 1985 and 2018 that was more pronounced in SCCs than BCCs, and for females than males. The highest AAPC occurred in the earliest periods of the study and started decreasing in the early 1990s for BCCs and in the late 1990s for SCCs. In the mid-2010s, there was suggestion of another change-point as age-standardised incidence rates started to plateau and decline, a change in trend that was statistically significant for first SCCs for females (Fig. 1a, b and Table 2).

**Table 2:** Age-adjusted average annual percentage change (AAPC) and 95% confidence intervals (CI) in the incidence rates of first (1985–2018) and annual (1978–2018) keratinocyte carcinomas, by histological type and sex^**a**^

Basal cell carcinoma				Squamous cell carcinoma			
First incidence^b^		Annual incidence^c^		First incidence^b^		Annual incidence^c^	
Period	AAPC (95% CI)	Period	AAPC (95% CI)	Period	AAPC (95% CI)	Period	AAPC (95% CI)
**Males**							
1985–1992	7.3 (4.5, 10.2)	1978–1984	7.2 (3.5, 11.1)	1985–1997	9.3 (8.0, 10.7)	1978–1981	− 1.6 (− 17.6, 17.4)
1992–2014	1.1 (0.7, 1.4)	1984–1988	16.8 (8.3, 25.9)	1997–2004	− 1.8 (− 4.1, 0.5)	1981–1998	11.4 (10.6, 12.3)
2014–2018	− 1.7 (− 5.2, 1.9)	1988–1997	7.3 (6.1, 8.5)	2004–2014	2.9 (1.7, 4.0)	1998–2001	− 3.4 (− 13.5, 7.8)
		1997–2014	2.6 (2.3, 2.9)	2014–2018	− 2.8 (− 6.0, 0.5)	2001–2014	3.2 (2.6, 3.7)
		2014–2018	− 1.2 (− 2.8, 0.5)			2014–2018	− 0.3 (− 2.6, 2.1)
1985–2018	2.0 (1.3, 2.8)	1978–2018	5.3 (4.3, 6.3)	1985–2018	3.4 (2.6, 4.3)	1978–2018	5.3 (3.7, 7.0)
**Females**							
1985–1988	15.8 (4.6, 28.1)	1978–1984	3.8 (− 0.7, 8.4)	1985–1996	13.4 (11.5, 15.4)	1978–1981	− 5.9 (− 25.3, 18.7)
1988–1997	4.2 (2.6, 5.9)	1984–1987	23.9 (1.8, 50.9)	1996–2011	1.5 (0.9, 2.2)	1981–1997	15.1 (13.9, 16.3)
1997–2016	1.6 (1.2, 2.0)	1987–1997	7.4 (6.1, 8.6)	2011–2014	6.3 (− 5.5, 19.6)	1997–2006	2.3 (1.0, 3.6)
2016–2018	− 7.2 (− 16.0, 2.6)	1997–2015	3.4 (3.1, 3.7)	2014–2018	− 4.2 (− 7.6, − 0.6)	2006–2014	4.4 (3.1, 5.7)
		2015–2018	− 4.0 (− 7.1, − 0.7)			2014–2018	− 1.7 (− 4.2, 0.9)
1985–2018	3.0 (1.8, 4.1)	1978–2018	5.3 (3.6, 7.0)	1985–2018	5.1 (3.8, 6.4)	1978–2018	6.6 (4.7, 8.5)
**Persons**							
1985–1988	14.4 (5.9, 23.6)	1978–1984	6.2 (2.8, 9.7)	1985–1996	11.6 (10.3, 13.0)	1978–1984	5.3 (− 0.2, 11.1)
1988–1996	3.8 (2.3, 5.5)	1984–1988	17.8 (9.8, 26.3)	1996–2005	0.1 (− 1.2, 1.3)	1984–1997	13.3 (12.1, 14.5)
1996–2016	1.3 (1.0, 1.6)	1988–1997	7.1 (6.0, 8.2)	2005–2014	3.1 (2.0, 4.2)	1997–2005	1.5 (0.1, 2.8)
2016–2018	− 5.9 (− 13.0, 1.9)	1997–2014	3.1 (2.8, 3.3)	2014–2018	− 3.0 (− 5.7, − 0.2)	2005–2014	4.0 (3.0, 4.9)
		2014–2018	− 1.5 (− 3.0, 0.1)			2014–2018	− 1.0 (− 3.1, 1.2)
1985–2018	2.6 (1.7, 3.5)	1978–2018	5.4 (4.5, 6.3)	1985–2018	4.2 (3.6, 4.9)	1978–2018	6.1 (5.1, 7.0)

^a^Different joinpoints were allowed by histological type and sex and Bayesian Information Criteria were used to select the optimal models (up to 5 joinpoints per model)

^b^Only first notification per person was included in the first counts, assuming that KCs registered from 1985 onwards without a previous history of registration by the Tasmanian Cancer Registry were the first ever KC notification for a person

^c^One notification per person per year was included in the annual counts

**Fig. 2:**
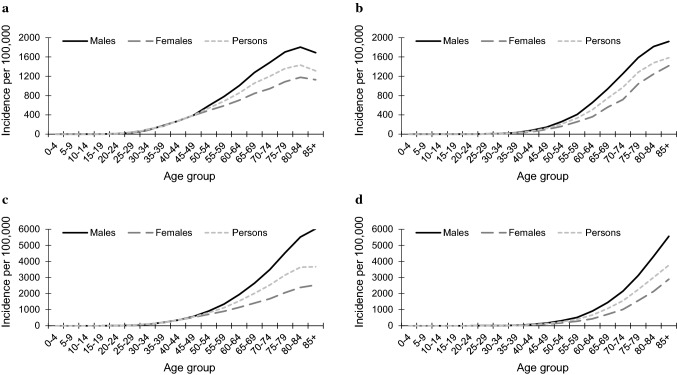
Age-specific incidence rates of keratinocyte carcinoma in Tasmania, by histological type and sex. **a** First basal cell carcinoma, 1985–2018; **b** first squamous cell carcinoma, 1985–2018; **c** annual basal cell carcinoma, 1978–2018; **d** annual squamous cell carcinoma, 1978–2018

Age-specific incidence rates increased with age and peaked at 80–84 years for BCCs and at 85 + years for SCCs. Incidence rates of BCCs increased from ages 35–39 years and more markedly for males after the age of 50 years. Incidence rates of SCCs increased from ages 45–49 years, and more prominently for males than females (Fig. 2a, b). Between 1985 and 2018, incidence rates of first KCs increased across all age groups. This increase appeared to have a negative linear relationship with age for BCCs for males and for SCCs for females, with the highest AAPC found in the younger age groups (Online Resource 4).

### Trends in the incidence rates of annual KCs

A total of 83,536 people were registered with a first and/or a subsequent KC notification between 1978 and 2018, of which 22% were registered with both BCCs and SCCs. Among people with a first BCC, 30% had more BCCs registered in multiple years (i.e., two or more); among people with a first SCC, 16% had more SCCs registered in multiple years. Of annual notifications, 65% were BCCs and 35% were SCCs (Table 1).

Age-standardised incidence rates of annual KCs increased more substantially than those of first KCs during the study period, with this increase more pronounced in SCC trends for females. Similar changes in trend that were described for incidence rates of first KCs were observed for annual KCs. A statistically significant change in trend from average annual percentage increase to decline was found in 2015 in incidence rates of BCC for females (Fig. 1c, d and Table 2).

Age-specific incidence rates of annual KCs followed similar patterns to those of first KCs, albeit they were more than twice as high in the older age groups, and especially for males (Fig. 2c, d). Between 1978 and 2018, incidence rates of annual KCs increased across all age groups. This increase appeared to have a positive linear relationship with age in BCC rates for females and in SCC rates for males, with the highest AAPC observed in the older age groups (Online Resource 5).

In the sensitivity analysis, excluding registrations within 3 months of an index notification resulted in slightly lower incidence rates of annual KCs than those found in the main analysis, but temporal and age-specific trends were unaffected (Online Resources 6 and 7).

### Cumulative risk

In 2018, the cumulative risk of developing a first KC was higher for BCCs than SCCs and for males than females (Table 3). In terms of lifetime risk, by age 80 years in Tasmania, one in 3.3 people were estimated to be diagnosed with a BCC, one in 4.3 people with a SCC, and one in 2.6 people with either histological type.

**Table 3:** Cumulative risk of being diagnosed with a histologically confirmed keratinocyte carcinoma by ages 75, 80 and 85 years in 2018, by histological type and sex

	Cumulative risk		
	Males	Females	Persons
**Basal cell carcinoma**			
By age 75 years	28%	23%	25%
By age 80 years	34%	28%	31%
By age 85 years	39%	32%	36%
**Squamous cell carcinoma**			
By age 75 years	20%	13%	16%
By age 80 years	28%	19%	23%
By age 85 years	34%	24%	29%
**Keratinocyte carcinoma (total)**			
By age 75 years	35%	29%	32%
By age 80 years	42%	35%	39%
By age 85 years	48%	41%	45%

## Discussion

This study describes temporal trends in incidence rates of histologically confirmed BCCs and SCCs over 41 years for the entire population of Tasmania. With high accuracy and ascertainment, the dataset compiled by the TCR is a reliable source of KC trend data in the state.

Age-standardised incidence rates of first and annual KCs increased over the study period; however, this increase was steeper in SCCs than BCCs, for females than males, and in annual than first KCs. For BCCs, the late 1980s was the period of highest increase, while for SCCs this period was extended into the mid-1990s. Since then, the AAPC has been declining and a change-point around 2014 indicates that incidence rates may have started to plateau and decline. This pattern was observed in all but a few age groups.

The most notable finding is that, despite a substantial increase over the study period, incidence rates of BCC and SCC have plateaued in Tasmania. Further, incidence rates of annual BCC and first SCC for females have declined since 2014. This is an encouraging finding and suggests that the sun awareness campaigns that started in the 1980s were effective. Other possible explanations, such as decreasing access to services, are unlikely given the growing number of skin cancer clinics established in Australia over the last decade and that skin cancers are commonly treated by general practitioners in primary care settings [25].

It was unexpected to find that younger age groups experienced some of the highest increase in the incidence of first KCs and substantial increase in the incidence of annual KCs. We assumed that younger people were the greatest beneficiaries of the sun awareness campaigns of the 1980s, as older people would have already suffered some of the damaging effects of sunlight exposure at the time. This finding is in contrast with those of previous studies in Australia that found declining incidence rates of KCs in the younger age groups [2, 11, 26], which suggests that different factors may be influencing sun exposure behaviours and/or the demographic profile of the people getting skin checks in Tasmania. Previous studies did not examine trends in the age-specific incidence of first KCs, so comparison is not possible.

Factual and artefactual factors may explain the rapid increase in incidence rates in the 1980s and 1990s. A factual increase was likely the result of changes in fashion experienced by the white-skinned Australian population throughout the 20th Century, when larger areas of the body became exposed to prolonged UV radiation, together with the growing popularity of tanning. While the use of solaria has been linked to increased skin cancer risk [27, 28], there are no data on their use in Australia during this earlier period, making it uncertain whether they contributed to this rapid increase. By contrast, rising awareness of skin cancer at the time may have led to an increasing number of people seeking medical attention and malignant lesions being detected.

The steeper increase in the incidence rates of annual than first KCs during the study period indicates that people with a first KC are having subsequent KCs detected in their lifetime. While it has been documented that those with a first KC are at an increased risk of developing further KCs [29, 30], detection bias may inflate these estimates because screening increases the incidence of KCs [31]. BCCs tend to develop slowly and can remain asymptomatic (i.e., without a history of irritation, bleeding, and ulceration) for extended periods, so those with previous diagnosed lesions are more likely to have new lesions detected due to increased screening. SCCs are more insidious but are still slow to develop into symptomatic lesions that would lead to investigation.

The slight delay in the decline of AAPCs in incidence rates of SCC compared with BCC in the 1980s and 1990s may be explained by differences in the age profile and sun exposure timing and patterns underlying the two histological types. Our finding that people with SCC have an older age profile than those with BCC is supported by the Australian literature [2, 11, 32, 33]. However, here we have demonstrated that the incidence rate of BCC peaks at ages 80 to 84 years while SCC continues to increase beyond the age of 85 years. BCC has been attributed to intermittent sunburns [34, 35], especially during early and middle life [36], while SCC has been attributed to continuous sun exposure and cumulative damage [37, 38]. This suggests that the adoption of sun smart behaviours could have a more immediate effect on incidence rates of BCC.

The reported incidence of annual KCs in Tasmania is the lowest in Australia, which is expected given the island’s latitude relative to the mainland. Previous studies have reported higher incidence rates for the southern Australian latitudes than those presented here [2, 8, 20, 33], but those estimates also included areas of the mainland that are closer to the equator. Differences in study methodology would also account for differences in results, as previous studies have relied on self-report measures or administrative data. Nonetheless, our findings support the latitude gradient previously reported in Australia [2, 8, 20, 33].

The incidence of KCs in Tasmania is high by international standards and more than twice as high as rates found in European countries [39–43] and Canada [44]. Tasmania is exposed to higher UV levels than these locations due to its, on average, slightly closer proximity to the equator and location in the Southern hemisphere, where air pollution levels are lower and the Earth is closer to the Sun due to its elliptical orbit [45].

The number of notifications in the TCR was higher than the Medicare claims for the surgical excision of KCs. This can be partly explained by the fact that Medicare does not include services provided in public hospitals and claims made to the Department of Veterans’ Affairs (an Australian Government department that provides services, including healthcare, to war veterans, members of the Australian Defence Force and the Australian Police Force and their families). These numbers further diverge after 2006 because the TCR’s automated coding system was unable to distinguish between biopsy samples and full excisions, resulting in the same lesion being registered twice in some instances. Our sensitivity analysis showed that excluding registrations within three months of an index notification did not markedly change the results.

This study shows that automated coding systems can provide a solution to the problem of KC registration in Australia. With less than 1% misclassification of histological type, the TCR system can be used to accurately track trends in the incidence of KCs. We are aware of one other system recently developed in Australia for this purpose [46]. In acknowledgment of the need for more accurate and reliable KC data, one of the solutions proposed by the Australian Institute of Health and Welfare was to mandate data collection by cancer registries [3]. The support of technology and automated coding systems will be paramount to the timely registration and reporting of KCs if data collection gains a legislative basis in Australia.

Our study has limitations. First, it only includes histologically confirmed KCs. Medicare requires that specimens be sent for histological confirmation before items are billed [47]. Yet in practice, lesions treated using destructive therapies, such as cryotherapy, are common and a specimen is not always sent for histological confirmation. Consequently, we have likely underestimated the incidence of KCs in Tasmania. Second, some Tasmanian residents may have had a KC diagnosis in another jurisdiction. Third, due to differences in UV exposure and climate between Tasmania and mainland Australia, point estimates cannot be generalised to the wider Australian population. Further, due to non-standardised reporting by notifiers and the limitations of our automated coding system, we were restricted to capturing information on one of each lesion type if multiple lesions were reported in a notification. We do not have information on the total number of lesions reported in each notification and cannot estimate the total burden of KCs in consequence. Nonetheless, the person-based incidence rates we report will assist health planners and policy makers monitor trends, identify high-risk demographic groups, develop targeted preventive strategies, and assess the effectiveness of public health campaigns.

This study has many strengths. First, it uses 41 years of population-level data, which has enabled the most comprehensive KC trend analysis in Australia to date. Second, including only histologically confirmed lesions eliminates error and recall bias that can occur in population surveys involving self-report of KC diagnoses. Third, cancer registry data have a significant advantage over Medicare data because BCCs and SCCs can be examined separately. And lastly, our large population-level dataset enabled us to derive robust estimates of incidence rates by histological type, sex, and age.

In conclusion, this study has shown that the TCR is a reliable source of KC trend data for the Tasmanian population. Incidence rates have increased substantially over the past four decades, and more markedly for annual than first KCs, SCCs than BCCs, and for females than males. More recently, incidence rates have started to plateau or decline, an encouraging finding that is likely a reflection of the effectiveness of sun awareness campaigns in Australia. However, with almost half of Tasmanians still being affected by age 80, it is necessary that programs continue to promote sun smart practices and the importance of skin cancer detection in this population.

## Supplementary Information

Below is the link to the electronic supplementary material.

Supplementary file 1 (PDF 32 KB)

Supplementary file 2 (PDF 55 KB)

Supplementary file 3 (PDF 38 KB)

Supplementary file 4 (PDF 59 KB)

Supplementary file 5 (PDF 59 KB)

Supplementary file 6 (PDF 70 KB)

Supplementary file 7 (PDF 72 KB)

## Data Availability

The data that support the findings of this study are available from the Tasmanian Cancer Registry, but restrictions apply to the availability of these data, which were used under licence for the current study, and so are not publicly available.

## Code availability

Joinpoint Regression Program can be downloaded from the National Cancer Institute, Division of Cancer Control & Population Sciences website (https://surveillance.cancer.gov/joinpoint/).
