# Supplementary material for: Temporal trends in the incidence rates of keratinocyte carcinomas from 1978 to 2018 in Tasmania, Australia: a population-based study
Source: Discov Oncol. 2021 Aug 31;12:30. doi: 10.1007/s12672-021-00426-5 (PMC8777529; doi:10.1007/s12672-021-00426-5)
Supplement: Supplementary file 1 — (PDF 32 KB) [file 12672_2021_426_MOESM1_ESM.pdf]

Online Resource 1 Percentage and number of misclassifications in the registration of keratinocyte carcinomas by the automated coding system in the sample of TeleForm™-processed paper notifications (n=1,000) and HL7 messages (n=1,500), by histological type

	**Misclassification, % (n)**			
	**Basal cell carcinoma**		**Squamous cell carcinoma**	
	**False negative**	**False positive**	**False negative**	**False positive**
TeleForm™	0.4% (4)	0.5% (5)	0.0% (0)	0.5% (5)
HL7 messages	0.1% (1)	0.1% (1)	0.0% (0)	0.1% (13)
Total	0.2% (5)	0.2% (6)	0.0% (0)	0.7% (18)
