# Supplementary material for: Temporal trends in the incidence rates of keratinocyte carcinomas from 1978 to 2018 in Tasmania, Australia: a population-based study
Source: Discov Oncol. 2021 Aug 31;12:30. doi: 10.1007/s12672-021-00426-5 (PMC8777529; doi:10.1007/s12672-021-00426-5)
Supplement: Supplementary file 2 — (PDF 55 KB) [file 12672_2021_426_MOESM2_ESM.pdf]

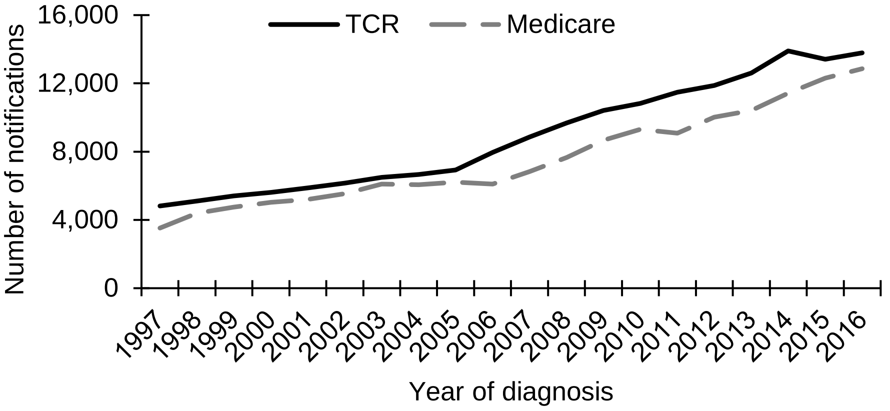

Online Resource 2 Comparison of the number of keratinocyte carcinoma notifications in the Tasmanian Cancer Registry with Medicare item numbers associated with the surgical excision of histologically confirmed keratinocyte carcinomas in Tasmania
