# Supplementary material for: Temporal trends in the incidence rates of keratinocyte carcinomas from 1978 to 2018 in Tasmania, Australia: a population-based study
Source: Discov Oncol. 2021 Aug 31;12:30. doi: 10.1007/s12672-021-00426-5 (PMC8777529; doi:10.1007/s12672-021-00426-5)
Supplement: Supplementary file 3 — (PDF 38 KB) [file 12672_2021_426_MOESM3_ESM.pdf]

Online Resource 3 Comparison of the age-standardised[a] incidence rates of annual keratinocyte carcinomas in the Tasmanian Cancer Registry for Tasmania[b] and in the National Non-Melanoma Skin Cancer Survey for Southern latitudes above 37°S [20]

	**1985**	**1990**	**1995**	**2002**
	**Tasmanian Cancer Registry**			
**Basal cell carcinoma**				
Males	179 (163, 196)	291 (271, 311)	437 (414, 460)	560 (536, 585)
Females	85 (74, 97)	168 (153, 183)	256 (238, 274)	350 (331, 370)
Persons	129 (119, 139)	226 (213, 238)	339 (324, 353)	448 (432, 463)
**Squamous cell carcinoma**				
Males	78 (67, 89)	126 (114, 139)	231 (215, 248)	270 (253, 286)
Females	29 (23, 35)	46 (39, 53)	102 (92, 113)	143 (131, 155)
Persons	52 (46, 58)	82 (75, 89)	161 (152, 171)	202 (192, 212)
	**National Non-Melanoma Skin Cancer Survey**			
**Basal cell carcinoma**				
Males	359 (223, 578)	421 (309, 532)	511 (398, 659)	646 (505, 827)
Females	307 (182, 519)	230 (151, 309)	360 (267, 486)	462 (349, 614)
Persons	333 (234, 474)	323 (256, 390)	430 (355, 522)	547 (454, 659)
**Squamous cell carcinoma**				
Males	64 (21, 199)	80 (35, 125)	229 (157, 334)	306 (217, 433)
Females	43 (11, 174)	13 (0, 30)	114 (66, 197)	171 (109, 267)
Persons	53 (22, 127)	47 (23, 71)	167 (123, 228)	232 (177, 306)

[a]Standardised to the 1960 World population.
[b]Tasmania is located between 40°S and 43°S.
