# Supplementary material for: Temporal trends in the incidence rates of keratinocyte carcinomas from 1978 to 2018 in Tasmania, Australia: a population-based study
Source: Discov Oncol. 2021 Aug 31;12:30. doi: 10.1007/s12672-021-00426-5 (PMC8777529; doi:10.1007/s12672-021-00426-5)
Supplement: Supplementary file 4 — (PDF 59 KB) [file 12672_2021_426_MOESM4_ESM.pdf]

Online Resource 4 Age-specific average annual percentage change (AAPC) and 95% confidence intervals (CI) in the incidence rates of first keratinocyte carcinomas[a] (1985-2018), by histological type and sex[b]

	Basal cell carcinoma				Squamous cell carcinoma			
	Males		Females		Males		Females	
**Age (years)**	**Period**	**AAPC (95% CI)**	**Period**	**AAPC (95% CI)**	**Period**	**AAPC (95% CI)**	**Period**	**AAPC (95% CI)**
**0-49**	1985-1989	17.5 (6.8, 29.3)	1985-1995	8.8 (6.6, 10.9)	1985-1987	-21.6 (-59.0, 49.9)	1985-1998	14.1 (9.7, 18.6)
	1989-2003	3.0 (1.7, 4.3)	1995-2016	1.7 (1.2, 2.2)	1987-1997	16.7 (11.3, 22.4)	1998-2018	1.7 (0.5, 3.0)
	2003-2018	-0.2 (-1.1, 0.8)	2016-2018	-13.4 (-28.7, 5.1)	1997-2011	-0.8 (-2.6, 1.1)		
					2011-2015	10.2 (-7.6, 31.5)		
					2015-2018	-18.5 (-33.4, -0.2)		
	1985-2018	3.2 (1.8, 4.5)	1985-2018	2.8 (1.5, 4.1)	1985-2018	2.2 (-2.6, 7.3)	1985-2018	6.4 (4.7, 8.2)
**50-59**	1985-1992	7.4 (3.1, 11.8)	1985-1996	6.1 (2.9, 9.5)	1985-1996	11.1 (7.5, 14.8)	1985-1997	16.3 (12.1, 20.8)
	1992-2018	1.2 (0.8, 1.5)	1996-2018	1.5 (0.9, 2.2)	1996-2018	1.2 (0.5, 1.8)	1997-2000	-9.9 (-36.8, 28.5)
							2000-2014	4.1 (2.5, 5.8)
							2014-2018	-5.7 (-13.7, 3.0)
	1985-2018	2.5 (1.6, 3.4)	1985-2018	3.1 (2.0, 4.2)	1985-2018	4.4 (3.2, 5.6)	1985-2018	5.7 (2.0, 9.5)
**60-69**	1985-1992	8.1 (4.7, 11.5)	1985-1988	18.8 (-4.0, 46.9)	1985-1988	-2.3 (-19.6, 18.6)	1985-1997	14.1 (11.6, 16.6)
	1992-2000	0.1 (-1.9, 2.3)	1988-2015	2.7 (2.2, 3.2)	1988-1995	12.7 (7.1, 18.6)	1997-2010	1.2 (-0.1, 2.5)
	2000-2016	2.3 (1.6, 3.1)	2015-2018	-6.1 (-14.2, 2.7)	1995-2006	-0.5 (-2.3, 1.3)	2010-2013	11.3 (-5.5, 31.2)
	2016-2018	-7.5 (-18.6, 5.0)			2006-2010	7.3 (-2.6, 18.3)	2013-2018	-3.9 (-7.2, -0.6)
					2010-2018	-0.4 (-2.2, 1.4)		
	1985-2018	2.3 (1.1, 3.5)	1985-2018	3.2 (1.1, 5.3)	1985-2018	3.0 (0.6, 5.4)	1985-2018	5.8 (3.9, 7.6)
**70-79**	1985-1994	7.6 (4.2, 11)	1985-1997	6.1 (4.1, 8.1)	1985-1998	9.0 (6.9, 11.1)	1985-1996	13.6 (10.6, 16.6)
	1994-2018	0.4 (-0.1, 0.9)	1997-2018	1.0 (0.5, 1.6)	1998-2002	-5.9 (-17.5, 7.3)	1996-2018	1.2 (0.7, 1.7)
					2002-2018	2.1 (1.2, 3.0)		
	1985-2018	2.3 (1.4, 3.2)	1985-2018	2.8 (2.1, 3.6)	1985-2018	3.7 (2.0, 5.5)	1985-2018	5.2 (4.2, 6.1)
**80+**	1985-1988	20.6 (-9.3, 60.4)	1985-1987	34.0 (-21.1, 127.5)	1985-1995	9.8 (5.4, 14.3)	1985-1995	13.2 (8.4, 18.2)
	1988-2018	0.1 (-0.4, 0.6)	1987-2005	2.7 (1.5, 3.8)	1995-2018	0.6 (0.0, 1.2)	1995-2018	1.5 (0.8, 2.1)
			2005-2018	-0.2 (-1.4, 1.0)				
	1985-2018	1.8 (-0.7, 4.4)	1985-2018	3.2 (0.0, 6.5)	1985-2018	3.3 (2.1, 4.6)	1985-2018	4.9 (3.5, 6.3)

[a]Only first notification per person was included in the first counts, assuming that KCs registered from 1985 onwards without a previous history of registration by the Tasmanian Cancer Registry were the first ever KC notification for a person.

[b]Different joinpoints were allowed by histological type and sex and Bayesian Information Criteria were used to select the optimal models (up to 5 joinpoints per model).
