# Supplementary material for: Temporal trends in the incidence rates of keratinocyte carcinomas from 1978 to 2018 in Tasmania, Australia: a population-based study
Source: Discov Oncol. 2021 Aug 31;12:30. doi: 10.1007/s12672-021-00426-5 (PMC8777529; doi:10.1007/s12672-021-00426-5)
Supplement: Supplementary file 5 — (PDF 59 KB) [file 12672_2021_426_MOESM5_ESM.pdf]

Online Resource 5 Age-specific average annual percentage change (AAPC) and 95% confidence intervals (CI) in the incidence rates of annual keratinocyte carcinomas[a] (1978-2018), by histological types and sex[b]

	Basal cell carcinoma				Squamous cell carcinoma			
	Males		Females		Males		Females	
**Age**	**Period**	**AAPC (95% CI)**	**Period**	**AAPC (95% CI)**	**Period**	**AAPC (95% CI)**	**Period**	**AAPC (95% CI)**
**0-49**	1978-1989	15.7 (12.9, 18.5)	1978-1995	11.5 (10.1, 12.9)	1978-1988	0.6 (-5.4, 6.9)	1978-1998	13.1 (10.4, 15.9)
	1989-1998	5.8 (3.4, 8.2)	1995-2016	2.2 (1.6, 2.7)	1988-1998	15.3 (9.8, 21.0)	1998-2018	2.0 (0.9, 3.3)
	1998-2011	1.5 (0.5, 2.6)	2016-2018	-13.3 (-29.2, 6.2)	1998-2018	0.0 (-1.1, 1.1)		
	2011-2018	-1.2 (-3.5, 1.1)						
	1978-2018	5.7 (4.7, 6.7)	1978-2018	5.2 (4.0, 6.4)	1978-2018	3.8 (1.8, 5.8)	1978-2018	7.4 (6.0, 8.8)
**50-59**	1978-1997	7.4 (6.5, 8.3)	1978-1995	10.1 (8.4, 11.8)	1978-1989	5.4 (0.9, 10.0)	1978-1997	14.6 (12.3, 17.1)
	1997-2015	2.1 (1.5, 2.6)	1995-2014	3.4 (2.7, 4.0)	1989-1996	14.1 (6.6, 22.2)	1997-2011	1.5 (0.0, 3.0)
	2015-2018	-3.3 (-9.9, 3.8)	2014-2018	-3.5 (-8.3, 1.6)	1996-2018	1.5 (0.9, 2.1)	2011-2014	13.9 (-8.1, 41.2)
							2014-2018	-8.9 (-15.3, -2.0)
	1978-2018	4.1 (3.4, 4.9)	1978-2018	5.5 (4.6, 6.4)	1978-2018	4.7 (2.9, 6.4)	1978-2018	7.3 (5.2, 9.5)
**60-69**	1978-1992	10.8 (9.5, 12.1)	1978-1995	9.7 (8.1, 11.3)	1978-1997	10.4 (9.3, 11.4)	1978-1984	0.6 (-7.8, 9.8)
	1992-2016	3.2 (3.0, 3.5)	1995-2015	4.2 (3.6, 4.8)	1997-2001	-2.9 (-11.7, 6.9)	1984-1999	14.3 (12.7, 15.9)
	2016-2018	-5.6 (-13.5, 3.1)	2015-2018	-3.9 (-10.0, 2.7)	2001-2014	3.5 (2.6, 4.5)	1999-2008	0.4 (-1.3, 2.2)
					2014-2018	-1.7 (-5.5, 2.2)	2008-2013	6.4 (2.1, 10.9)
							2013-2018	-0.8 (-3.3, 1.7)
	1978-2018	5.4 (4.7, 6.0)	1978-2018	5.9 (5.0, 6.7)	1978-2018	5.5 (4.3, 6.7)	1978-2018	6.0 (4.4, 7.7)
**70-79**	1978-1993	12.8 (11.3, 14.3)	1978-1982	1.9 (-12.8, 19.1)	1978-1998	12.1 (11.0, 13.2)	1978-1996	15.7 (14.2, 17.3)
	1993-1999	6.3 (2.8, 9.9)	1982-1987	18.4 (6.3, 31.8)	1998-2002	-2.2 (-10.4, 6.7)	1996-2014	3.3 (2.8, 3.9)
	1999-2018	1.9 (1.6, 2.2)	1987-1998	7.9 (6.2, 9.7)	2002-2018	2.5 (2.0, 3.1)	2014-2018	-0.9 (-4.6, 3.0)
			1998-2016	3.7 (3.2, 4.2)				
			2016-2018	-4.7 (-14.0, 5.6)				
	1978-2018	6.5 (5.8, 7.3)	1978-2018	6.0 (3.8, 8.2)	1978-2018	6.7 (5.7, 7.8)	1978-2018	8.3 (7.5, 9.1)
**80+**	1978-1997	10.4 (8.7, 12.1)	1978-1992	12.7 (9.5, 16.0)	1978-1997	11.7 (10.0, 13.4)	1978-1996	14.8 (12.6, 17.0)
	1997-2010	4.1 (3.0, 5.3)	1992-2012	4.7 (4.0, 5.4)	1997-2014	3.6 (2.9, 4.3)	1996-2014	4.5 (3.8, 5.2)
	2010-2018	0.0 (-1.5, 1.5)	2012-2018	-0.4 (-3.1, 2.3)	2014-2018	0.4 (-3.3, 4.2)	2014-2018	-0.7 (-5.0, 3.9)
	1978-2018	6.2 (5.3, 7.1)	1978-2018	6.6 (5.5, 7.8)	1978-2018	7.0 (6.1, 7.9)	1978-2018	8.5 (7.4, 9.5)

[a]One notification per person per year was included in the annual counts.

[b]Different joinpoints were allowed by histological type and sex and Bayesian Information Criteria were used to select the optimal models (up to 5 joinpoints per model).
