# Supplementary material for: Temporal trends in the incidence rates of keratinocyte carcinomas from 1978 to 2018 in Tasmania, Australia: a population-based study
Source: Discov Oncol. 2021 Aug 31;12:30. doi: 10.1007/s12672-021-00426-5 (PMC8777529; doi:10.1007/s12672-021-00426-5)
Supplement: Supplementary file 6 — (PDF 70 KB) [file 12672_2021_426_MOESM6_ESM.pdf]

a

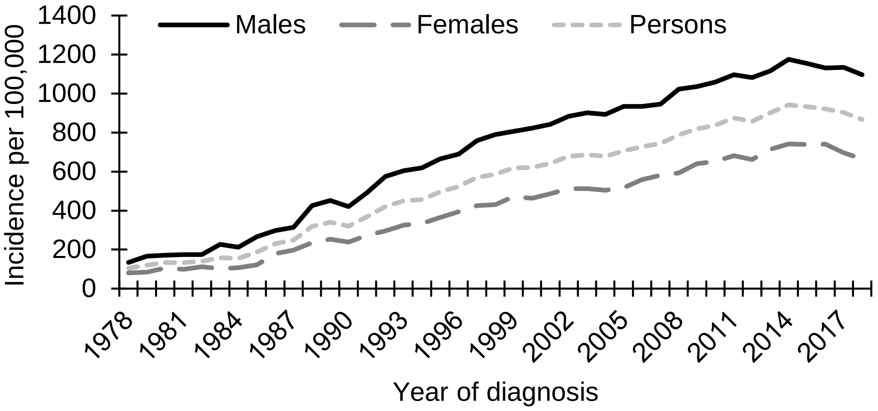

b

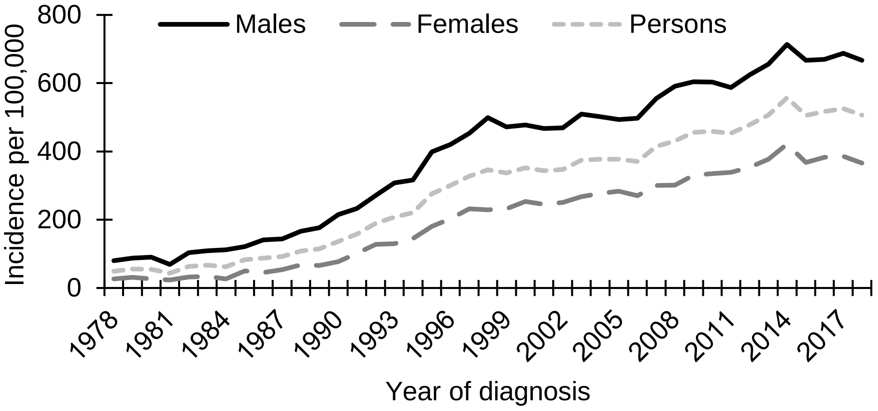

Online Resource 6 Temporal trends in the incidence rates of annual keratinocyte carcinoma in Tasmania, by histological type and sex, 1978-2018. Incidence rates are standardised to the Australian 2001 population. Registrations within three months of an index notification were excluded. **a**. basal cell carcinoma; **b.** squamous cell carcinoma
