# Supplementary material for: Temporal trends in the incidence rates of keratinocyte carcinomas from 1978 to 2018 in Tasmania, Australia: a population-based study
Source: Discov Oncol. 2021 Aug 31;12:30. doi: 10.1007/s12672-021-00426-5 (PMC8777529; doi:10.1007/s12672-021-00426-5)
Supplement: Supplementary file 7 — (PDF 72 KB) [file 12672_2021_426_MOESM7_ESM.pdf]

a

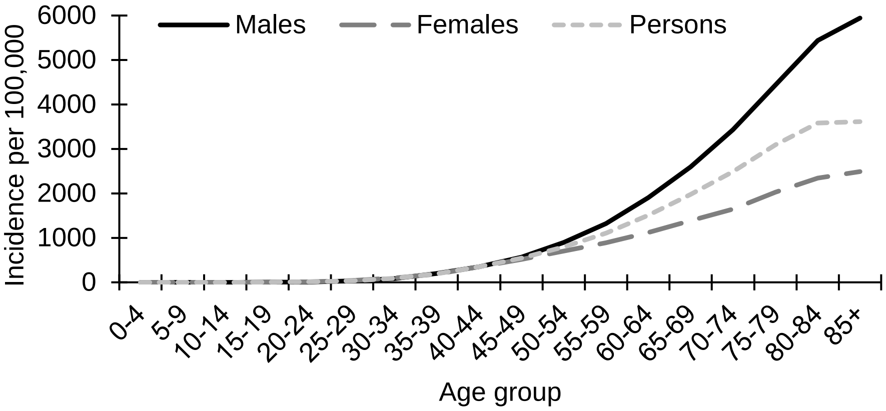

b

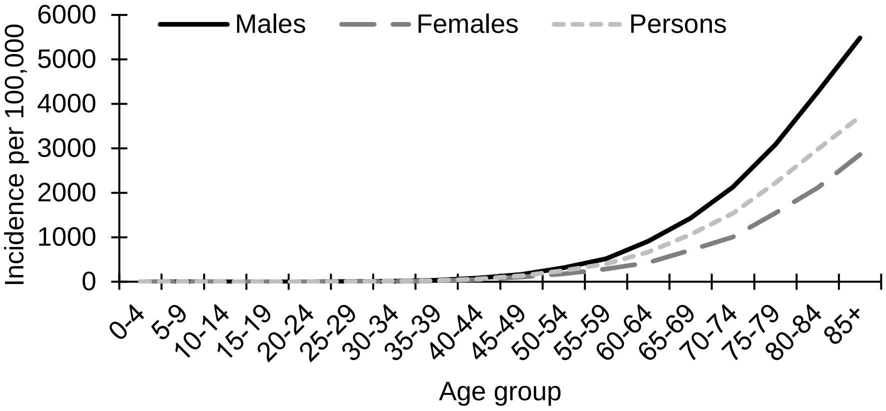

Online Resource 7 Age-specific incidence rates of keratinocyte carcinoma in Tasmania, by histological type and sex, 1978-2018. Registrations within three months of an index notification were excluded. **a**. basal cell carcinoma; **b.** squamous cell carcinoma
